# *In silico* analysis of the impact of toxic metals on COVID-19 complications: molecular insights

**DOI:** 10.2478/aiht-2024-75-3819

**Published:** 2024-06-29

**Authors:** Jovana Živanović, Katarina Baralić, Katarina Živančević, Dragica Božić, Đurđica Marić, Evica Antonijević Miljaković, Aleksandra Buha Đorđević, Marijana Ćurčić, Zorica Bulat, Biljana Antonijević, Danijela Đukić-Ćosić

**Affiliations:** University of Belgrade, Faculty of Pharmacy, Department of Toxicology “Akademik Danilo Soldatović”, Belgrade, Serbia; University of Belgrade, Faculty of Biology, Ivan Đaja Institute for Physiology and Biochemistry, Belgrade, Serbia

**Keywords:** Comparative Toxicogenomic Database, cytokines, genes, physical interactions, SARS-CoV-2, citokini, fizičke interakcije, geni, komparativna toksikogenomska baza podataka (CTD), SARS-CoV-2

## Abstract

COVID-19 can cause a range of complications, including cardiovascular, renal, and/or respiratory insufficiencies, yet little is known of its potential effects in persons exposed to toxic metals. The aim of this study was to answer this question with *in silico* toxicogenomic methods that can provide molecular insights into COVID-19 complications owed to exposure to arsenic, cadmium, lead, mercury, nickel, and chromium. For this purpose we relied on the Comparative Toxicogenomic Database (CTD), GeneMANIA, and ToppGene Suite portal and identified a set of five common genes (*IL1B, CXCL8, IL6, IL10, TNF*) for the six metals and COVID-19, all of which code for pro-inflammatory and anti-inflammatory cytokines. The list was expanded with additional 20 related genes. Physical interactions are the most common between the genes affected by the six metals (77.64 %), while the dominant interaction between the genes affected by each metal separately is co-expression (As 56.35 %, Cd 64.07 %, Pb 71.5 %, Hg 81.91 %, Ni 64.28 %, Cr 88.51 %). Biological processes, molecular functions, and pathways in which these 25 genes participate are closely related to cytokines and cytokine storm implicated in the development of COVID-19 complications. In other words, our findings confirm that exposure to toxic metals, alone or in combinations, might escalate COVID-19 severity.

## Sažetak

COVID-19 može izazvati niz komplikacija, uključujući kardiovaskularnu, bubrežnu i/ili respiratornu insuficijenciju, ali se malo zna o njegovim potencijalnim učincima u osoba koje su izložene toksičnim metalima. Cilj ovog istraživanja bio je odgovoriti na to pitanje pomoću *in silico* toksikogenomske metode, koja može pružiti molekularni uvid u komplikacije bolesti COVID-19 uslijed izloženosti arsenu, kadmiju, olovu, živi, niklu i kromu. U tu su svrhu korišteni Komparativna toksikogenomska baza podataka (CTD), GeneMANIA i ToppGene Suite portal te je identificirana skupina od pet zajedničkih gena (*IL1B, CXCL8, IL6, IL10, TNF*) za šest metala i COVID-19, koji svi kodiraju proinflamatorne i antiinflamatorne citokine. Lista je proširena s dodatnih 20 srodnih gena. Fizičke interakcije dominirale su između gena na koje utječe kombinacija ispitivanih metala (77,64 %), a koekspresija je dominantna interakcija između gena na koje djeluju pojedinačni metali (As 56,35 %, Cd 64,07 %, Pb 71,5 %, Hg 81,91 %, Ni 64,28 %, Cr 88,51 %). Biološki procesi, molekulske funkcije i putovi u kojima sudjeluje tih 25 gena blisko su povezani s citokinima i citokinskom olujom, koja je uključena u razvoj komplikacija bolesti COVID-19. Drugim riječima, ovi rezultati potvrđuju da izloženost toksičnim metalima, bilo pojedinačno ili u kombinaciji, može dovesti do razvoja težih oblika bolesti COVID-19.

Besides affecting the respiratory system and causing persistent respiratory conditions (1), the coronavirus disease 2019 (COVID-19) can lead to complications affecting the cardiovascular, renal, and neurological systems (2,3,4). Conversely, conditions like cardiovascular diseases, obesity, asthma, chronic obstructive pulmonary disease, diabetes, tumours, renal, neurological diseases, inflammation, coagulation disorders, and factors such as age, sex, and lifestyle also contribute to the severity of COVID-19 (5,6,7). Finally, the severity and resulting complications are believed to be aggravated by exposure to various environmental pollutants, toxic metals in particular (6, 8,9,10,11). However, the extent of their contribution remains uncertain.

According to the Agency for Toxic Substances and Disease Registry (ATSDR), the top list of these pollutants includes arsenic (As), cadmium (Cd), lead (Pb), and mercury (Hg) (9, 10). Moreover, the International Agency for Research on Cancer (IARC) has classified As, Cd, nickel (Ni), and chromium (Cr) as human carcinogens, and all four metals have been associated with the development of lung cancer (11,12,13,14).

The aim of our study was to take a look at likely molecular mechanisms underlying COVID-19 complications in the context of exposure to six toxic metals (As, Cd, Pb, Hg, Ni, and Cr) by employing toxicogenomic research databases that investigate the interplay between the genes involved in environmental stress and disease development (15). This kind of approach can help to identify and predict gene functions within specific pathways. Furthermore, it provides methods to examine diverse interactions involving chemicals, genes, proteins, and metabolites and to identify networks that could play a crucial role in causing specific harmful effects (16).

## METHODS

The relationships between the selected toxic metals and COVID-19 disease complications were investigated using the Comparative Toxicogenomics Database (CTD) (http://ctdbase.org/), which contains information on the relationships between the diseases, genes, and substances (17, 18). By browsing the CTD gene cards we identified the genes interacting with each of the investigated toxic metals. Then, using the “disease” tabs in MyVenn CTD tool we obtained a list of genes related to the development of COVID-19 complications associated with these toxic metals. The next step was to analyse the effects of these toxic metals on the obtained genes using the “gene interactions” CTD tabs. Only binary interactions were considered, that is, those between a single chemical and a single gene. Complex interactions, such as the effect of numerous substances on the expression of one or multiple genes, were not considered.

All results presented in this article are based on data available at the time of analysis (September 2023), and the number of genes associated with the selected metals and COVID-19 complications may have changed since, as the database is updated constantly.

The gene set identified with the CTD analysis was then entered into the GeneMANIA prediction tool (*http://genemania.org*) to retrieve relevant related genes and comprehensive information regarding their interactions with the original set of genes (19,20,21).

**Figure 1:**
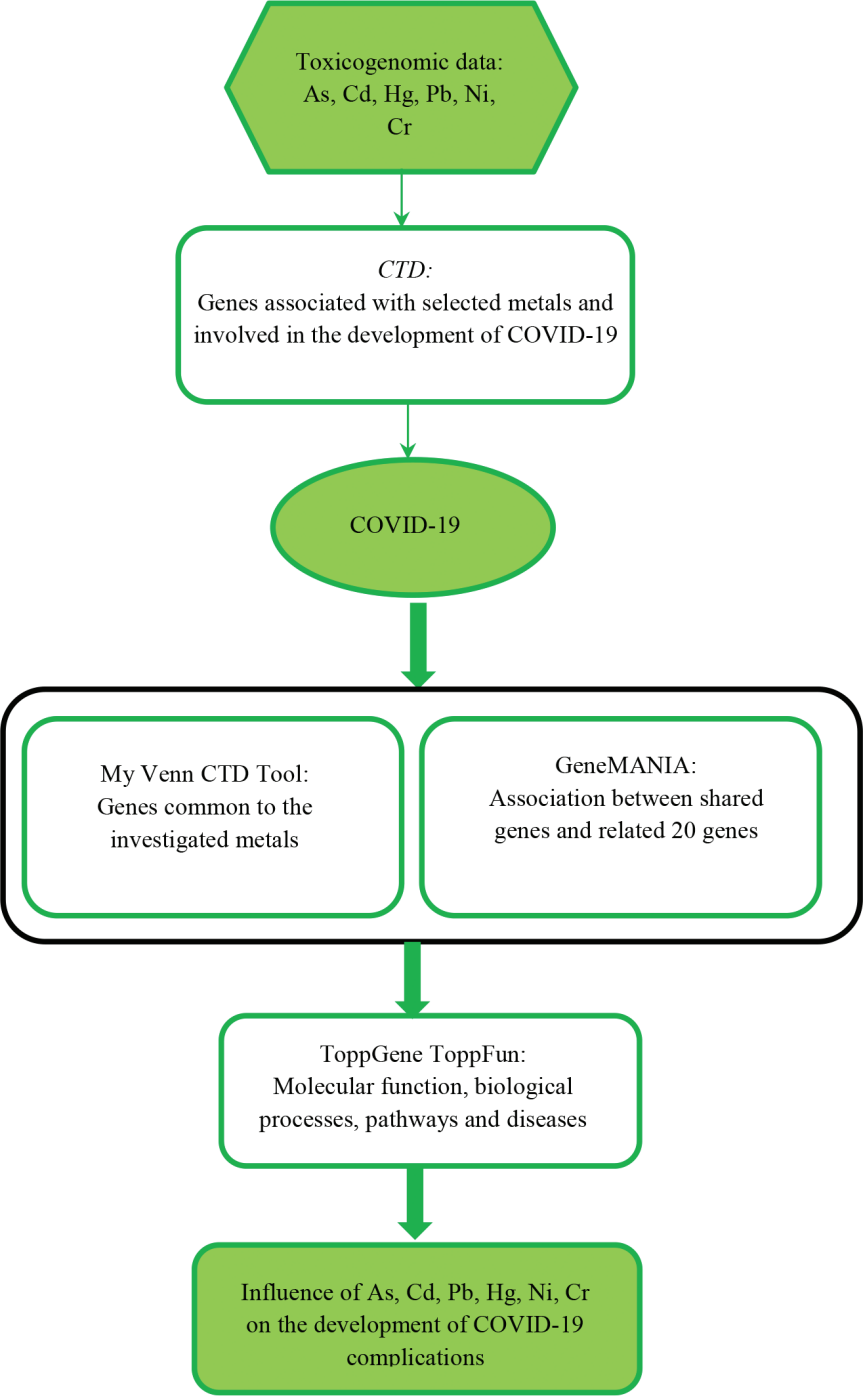
Flow chart of the steps used to analyse the association between exposure to toxic metals and COVID-19 complications

To further explore the molecular mechanisms associated with exposure to toxic metals and development of COVID-19 complications we used the ToppGene ToppFun function (p<0.05, false discovery rate-corrected) (22). The input gene set comprised the five common genes obtained with the CTD analysis and 20 related genes obtained with the GeneMANIA tool. Information on molecular functions, biological processes, molecular pathways, and diseases potentially contributing to the development of COVID-19 complications was ranked based on the p-value. The number of genes involved in each process was determined, and the top five molecular functions, biological processes, molecular pathways, and diseases were listed. A detailed flow chart of the process is presented in Figure 1.

## RESULTS AND DISCUSSION

Our CTD research revealed five genes – namely *CXCL8, IL10, IL1B, IL6*, and *TNF* – to be affected by all the investigated toxic metals (Table 1). Interleukin 1B (IL1B) is a pro-inflammatory cytokine that plays a role in a variety of inflammatory lung diseases. Increased levels can cause an overreaction of the immune system resulting in excessive production of reactive oxygen and nitrogen species (ROS and RNS) (23). In a recent study (24), an IL1B receptor antagonist, anakinra, was intravenously administered to a group of patients. After 21 days, notable improvements in respiratory function and a decrease in the level of C-reactive protein, a key indicator of inflammation, were observed compared to the patients who did not receive this treatment.

**Table 1:** Genes associated with COVID-19 and the investigated metals (*CTD: https://ctdbase.org/*)

**Toxic metals**	**Total number of genes**	**Genes associated with COVID-19**
Arsenic (As)	4997	*ATP11A, BCL11A, CCL2, CCL3, CCR2, CRP, CXCL8, ICAM5, IFNAR2, IL10, IL10RB, IL1B, IL2, IL2RA, IL6, IL7, LZTFL1, MUC1, OAS1, TNF*
Cadmium (Cd)	5281	*AGT, CCL2, CCR2, CD209, CRP, CXCL8, F8, HCK, IL10, IL1B, IL2, IL6, IL7, MUC1, OAS1, SELE, TNF, UGCG*
Lead (Pb)	3278	*AGT, BSG, CCL2, CCL3, CD209, CRP, CXCL10, CXCL8, ICAM5, IL10, IL1B, IL6, TNF*
Mercury (Hg)	635	*CCL3, CCR2, CRP, CXCL8, IL10, IL1B, IL2, IL6, TNF, UGCG*
Nickel (Ni)	7665	*ACE2, AGT, BCL11A, BTK, CCL2, CCL3, CCR2, CD209, CRP, CXCL10, CXCL8, FUT2, HCK, IFNAR2, IL10, IL10RB, IL1B, IL2, IL2RA, IL6, IL7, OAS1, PLSCR1, SELE, TMPRSS2, TMPRSS4, TNF*
Chromium (Cr)	2353	*AGT, CXCL8, IL10, IL1B, IL6, TNF, UGCG*
**As+Cd+Pb+Hg+Ni+Cr**	**5**	***CXCL8, IL10, IL1B, IL6, TNF***

Tumour necrosis factor (TNF) is another important inflammatory cytokine associated with excessive immune response (25) and inflammatory lung conditions such as COVID-19. It triggers inflammatory and proteolytic pathways and interferes with processes responsible for controlling inflammatory response. Both serum TNF and IL6 levels are predictors of survival in COVID-19 patients. An overactive immune response caused by TNF, IL1B, and IL6 is the most common cause of COVID-19 complications and death (26).

Interleukin 10 (IL10) is an anti-inflammatory cytokine that, while safeguarding against various pathological conditions, paradoxically promotes lung fibrosis (27).

Interleukin 8, also known as CXCL8, is a neutrophil-attracting chemokine. High levels have been reported in bronchial epithelial cells of patients infected with the corona virus (28).

COVID-19 is marked by elevated cytokine levels in both blood and epithelial lining fluid obtained with bronchoalveolar lavage, reflecting the immune response to the virus. Alveolar macrophages and monocytes produce IL6, TNF, IL8, and IL1B, while neutrophils produce ROS, lipid mediators, and proteases, all of which are toxic to the virus but also to the lungs. Furthermore, IL6, TNF, IL8, IL1B, and IL10 levels have been reported to correlate with the clinical severity of COVID-19 (29, 30).

Table 2 details the interactions between each toxic metal and the five shared genes. Each of the six metals appears to upregulate *CXCL8* mRNA expression and IL8 levels, more so in patients with severe clinical manifestations of COVID-19 (28). As and Cd seem to both up- and downregulate *IL1B*, while Pb, Hg, and Ni upregulate *IL1B*. As, Pb, Ni, and Hg upregulate *IL6*, whereas Cd has a dual effect on *IL6* mRNA and upregulates protein expression. Pb, Hg, Ni, and Cr upregulate *TNF*, whereas As and Cd show both effects. Overall, our findings suggest that all the studied metals contribute to COVID-19 complications via one or more common genes.

**Table 2:** Effects of As, Cd, Pb, Hg, Ni, and Cr on common genes

**Toxic metals**	**Interactions**	***CXCL8***	***IL10***	***IL1B***	***IL6***	***TNF***
As	Expression of mRNA	N/A	↓	↓↑	↑	↓
	Protein expression	↑	↑	↓↑	↑	↓↑
Cd	Expression of mRNA	↑	↓	↓↑	↓↑	↓↑
	Protein expression	↑	↑	↑	↑	↑
Pb	Expression of mRNA	↑	N/A	↑	N/A	↑
	Protein expression	↑	↓↑	↑	↑	↑
Hg	Expression of mRNA	N/A	↑	↑	N/A	↑
	Protein expression	↑	N/A	↑	↑	↑
Ni	Expression of mRNA	↑	↑	↑	↑	↑
	Protein expression	↑	N/A	↑	↑	↑
Cr	Expression of mRNA	↑	N/A	N/A	N/A	↑
	Protein expression	N/A	N/A	N/A	↓	↑

↑ – upregulation; ↓ – downregulation; ↑↓ – upregulation and downregulation. N/A – no data available in CTD; *IL1B* – interleukin 1b; *IL6* – interleukin 6; *IL10* – interleukin 10; *CXCL8* – interleukin 8; *TNF* – tumour necrosis factor

**Figure 2:**
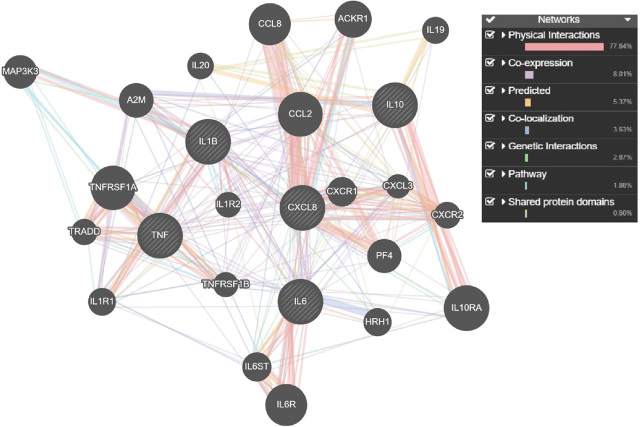
Network of five genes/proteins associated with the development of COVID-19 and affected by As, Cd, Pb, Hg, Ni, and Cr and their relationship with 20 genes/proteins, identified by the GeneMANIA prediction tool (*https://genemania.org*). Legend: pink lines – physical interactions; purple lines – co-expression; orange lines – server-predicted interactions; blue lines – co-localisation; green lines – genetic interactions; light blue lines – shared pathway; gray-yellow lines – shared protein domains

Figure 2 shows the interactions between all 25 genes identified by CTD and GeneMANIA (five input and 20 related genes) by interaction type: physical interactions (77.64 %), co-expression (8.01 %), server-predicted interactions (5.37 %), co-localisation (3.63 %), genetic interactions (2.87 %), shared pathway (1.88 %), and occurrences of shared protein domains (0.60 %). Physical interactions means that the genes are related if their proteins interact. Co-expression means that the genes are related if their expression is similar under test conditions. Server-predicted interactions means that extracted protein interactions are predicted based on known interactions between orthologous genes in other organisms. If two proteins interact in one organism (e.g. human), their orthologues are expected to interact in another (e.g. mouse). Co-localisation means that genes are expressed or their proteins are found in the same tissue. Genetic interactions means that a change in one gene triggers changes in another. Pathway interactions means that proteins coded by two genes participate in the same reaction pathway. Shared protein domains means that proteins coded by two genes have similar domains (19,20,21).

**Figure 3:**
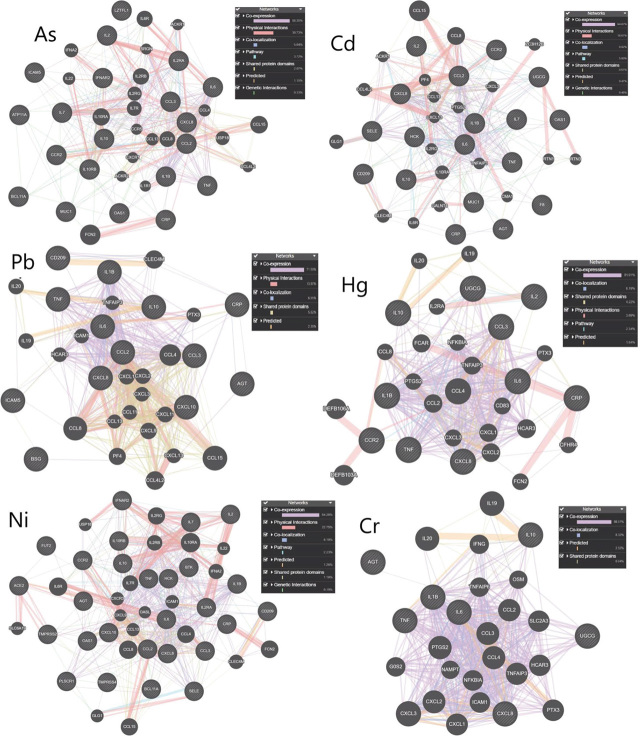
Networks of five genes/proteins associated with the development of COVID-19 and the 20 related genes for each toxic metal separately. Legend: pink lines – physical interactions; purple lines – co-expression; orange lines – server-predicted interactions; blue lines – co-localisation; green lines – genetic interactions; light blue lines – shared pathway; gray-yellow lines – shared protein domains

Figure 3 details interactions between genes linked to each metal separately and the nature of interactions among them. The dominant type of interaction is co-expression, whereas the interactions between the genes common to all investigated metals are mostly physical.

Interactions between the obtained sets of genes reveal how the molecular dynamics of these genes contribute to the exacerbation of COVID-19. We have chosen to present both the interactions between genes with which individual metals interact and the interactions between genes with which all the investigated metals interact. This decision was made with the understanding that examining interactions between genes and individual metals provide insights into metal-specific interactions. On the other hand, studying interactions with all investigated metals collectively sheds light on common interactions affected by combined metal exposure. This highlights the complex functions of genes shared among all the investigated metals and those unique to each metal, providing insights into the diverse molecular processes that may contribute to the severity of COVID-19 complications.

Table 3 shows the correlations between the 25 identified genes and various biological processes, molecular functions, pathways, and diseases contributing to the development of COVID-19 complications. It also lists the number of genes involved in each biological process, molecular function, pathway, and disease.

**Table 3:** Correlation of the 25 identified genes (five common and 20 related) with biological processes, molecular functions, pathways, and diseases associated with COVID-19 complications

**Category**	**Name**	**ID**	***p*-value**	Genes involved*
Molecular function	cytokine binding	GO:0019955	1.341E-17	11
	cytokine receptor binding	GO:0005126	2.170E-17	13
	cytokine activity	GO:0005125	3.580E-15	11
	signalling receptor binding	GO:0005102	1.187E-11	16
	receptor ligand activity	GO:0048018	1.952E-11	11
Biological process	inflammatory response	GO:0006954	4.830E-28	23
	cytokine-mediated signalling pathway	GO:0019221	1.992E-25	19
	cellular response to cytokine stimulus	GO:0071345	1.277E-20	19
	response to cytokine	GO:0034097	9.706E-20	19
	leukocyte migration	GO:0050900	8.016E-19	15
Pathway	KEGG cytokine receptor interaction	M9809	1.039E-30	20
	reactome interleukin 10 signalling	M27605	6.459E-22	11
	reactome signalling by interleukins	M874	4.294E-18	16
	WP SARSCOV2 innate immunity evasion and cell-specific immune response	M40067	9.207E-18	10
	WP overview of proinflammatory and profibrotic mediators	M42533	1.091E-16	11
Disease	susceptibility to HIV infection	609423	6.577E-6	3
	malaria, susceptibility to	cv:C1836230	6.577E-6	3
	white blood cell count quantitative trait locus 1	611162	5.893E-4	2
	WHIM syndrome 2	cv:C1970028	5.893E-4	2
	Graft-versus-host disease, susceptibility to	611862	2.333E-3	1

* Number of genes involved in each category. KEGG – Kyoto Encyclopedia of Genes and Genomes; WHIM – warts, hypogammaglobulinaemia, infections, and myelokathexis syndrome type 2; WP – WikiPathways

Relying on gene ontology and pathway analysis, our findings provide a comprehensive understanding of the intricate molecular and cellular mechanisms behind the interplay between toxic metal exposure and COVID-19 severity. Three molecular functions stand out: cytokine binding, cytokine binding to receptors, and cytokine activity. Similarly, the most common biological processes involve inflammatory response, cytokine-mediated signalling pathway, and cellular response to cytokine stimulation. All this suggests that the common genes for As, Cd, Pb, Hg, Ni, and Cr, as well as the 20 related genes are involved in the cytokine storm, a key event in the development of COVID-19 complications. Cytokine-cytokine receptor interaction, the IL10 signalling pathway, and interleukin signalling are the most important pathways in which the investigated genes are involved. The involvement of IL10, along with IL6, in cytokine-cytokine receptor interaction has already been reported in patients with severe clinical symptoms (31).

In patients with severe clinical manifestations of the disease, uncontrolled systemic inflammation and cytokine storm occur as a result of the excessive production of pro-inflammatory cytokines, which damages many tissues and can lead to their insufficiency, acute respiratory distress syndrome, sepsis-induced shock, and death. The cytokine storm can cause T-lymphocyte apoptosis or necrosis, weakening the organism’s defences against the pathogen (32). Cytokines facilitate communication between the immune and hematopoietic cells by binding to receptors on the target cell surface. Notably, a single cytokine can carry out diverse biological functions across different tissues and cell types (33). Patients with more severe clinical manifestations often have comorbidities such as diabetes, hypertension, and cardiovascular disease, which may weaken their capacity to tolerate systemic cytokines (34). Furthermore, IL1B and IL6 produced by infected tissue are involved in megakaryocyte function and platelet production, the consequence of which is hypercoagulation (35).

IL6 can affect cells via two signalling pathways: cis and trans. The cis signalling pathway involves IL6 binding to its immune cell membrane receptor complexed with *gp130* and signal transduction by Janus kinase (JAK) and signal transducer and activator of transcription 3 (STAT3). The trans signalling pathway is activated by IL6 binding to the receptor’s soluble form, which then forms a complex with the gp130 dimer present on almost all cell surfaces. Both processes culminate in a cytokine storm, triggering the release of the vascular endothelial growth factor (VEGF), monocyte chemoattractant protein 1 (MCP-1), IL8, and more IL6, while simultaneously reducing the expression of E-cadherin. High VEGF and low E-cadherin levels increase vascular permeability and ultimately contribute to the onset of pulmonary dysfunction in acute respiratory distress syndrome (ARDS) (36).

The existing evidence indicates that individuals with pre-existing health conditions are more susceptible to contracting COVID-19 and experiencing complications. This, in particular, concerns patients with HIV infection, whose HIV antiretroviral therapy has proved ineffective against SARS-CoV-2 infection (37, 38). In contrast, COVID-19 has a lower prevalence in endemic malarial areas. One of the reasons is that the residents of these low-income areas have fewer opportunities to test for SARS-CoV 2 infection due to limited resources, but also that COVID-19 may be misdiagnosed for malaria due to symptom similarities (39). It has been shown that the *ACE2* gene polymorphism (C1173T substitution and deletion/insertion polymorphism) responsible for SARS-CoV-2 entry and development of antibodies to glycoprotein 1 in malaria patients may recognize the SARS-CoV-2 glycoprotein and lower the risk of severe disease (40). For this reason, the antimalarial drug hydroxychloroquine has been incorporated into therapeutic protocols for COVID-19 treatment.

## CONCLUSION

Our study highlights the value of free online toxicogenomic data mining and analysis tools, such as CTD, ToppGene Suite, and GeneMANIA. However, they come with certain limitations, as they may not include all available information and miss specific interaction data (19). Furthermore, while these tools can statistically identify associations between genes affected by chemicals and those involved in environmental diseases such as SARS-CoV-2, they do not establish dose-response relationships or take into account various exposure factors which could affect the final outcome (16). Even so, they provide indirect access to literature, allowing users to gather information on factors like duration of exposure, dose, and exposure period (41). In other words, *in silico* toxicogenomic tools such as these cannot replace toxicological methods, but can certainly complement them and provide insights or hypotheses that can guide further research.

By applying parameters of interest, our study has revealed how exposure to toxic metals can aggravate COVID-19 and its complications, primarily by inducing changes in gene expression. Interactions between the five genes shared by all the investigated metals and COVID-19 (*CXCL8*, *IL1B*, *IL10*, *IL6*, and *TNF*) are mainly physical, whereas the dominant interaction between the genes affected by individual metals is co-expression. Gene ontology and pathway analysis points to the involvement of these genes in inflammatory, cytokine-related functions, crucial for understanding COVID-19 complications, especially in the context of the cytokine storm. This underscores the significant impact of environmental factors, such as toxic metals, on disease severity. Finally, this methodology proves valuable for investigating the molecular and cellular mechanisms of diseases associated with environmental chemicals.
